# Myocardial performance after coronary re-implantation in pediatric patients assessed with conventional echocardiographic and 2D-speckle tracking analysis: a case-control study

**DOI:** 10.11604/pamj.2021.38.29.26111

**Published:** 2021-01-13

**Authors:** Salma Charfeddine, Dorra Abid, Rania Hammami, Rania Gargouri, Leila Abid, Faten Triki, Samir Kammoun

**Affiliations:** 1 Cardiology Department, Hedi Chaker University Hospital, University of Medicine of Sfax, Sfax, Tunisia

**Keywords:** Coronary reimplantation, arterial switch operation, speckle-tracking echocardiography, ventricular function

## Abstract

**Introduction:**

reduced exercise capacity, coronary artery abnormalities and reversible myocardial ischemia have been demonstrated after arterial switch operation (ASO) and coronary reimplantation. Despite this, indices of systolic function, assessed by standard and Doppler echocardiography, are within the normal range. The aim of this study was to highlight the long-term changes in myocardial function following coronary reimplantation using Doppler and speckle-tracking imaging (STI) echocardiography.

**Methods:**

this observational case control study included 36 patients and 20 gender and age-matched healthy controls. A group study was performed using patients who were followed for at least 6 months after the operation and who visited the pediatric cardiology outpatient between October 2015 and May 2016. Systolic and diastolic parameters, left ventricle (LV) and right ventricle (RV) myocardial performance were assessed in each group.

**Results:**

the LV global peak strain parameters revealed a significant decrease in the longitudinal and circumferential strain components. The LV global longitudinal strain (GLS) values were lower in both groups of operated patients than controls (-19.9 ± 2.2% (group 1) versus -20.9 ± 1.6% (group 2) versus -22.9 ± 2.3% (group 3), p<0.001). The patients with coronary reimplantation had the lowest values. The LV global circumferential strain was also decreased in the group 1 patients as compared with the 2 other groups (-16.6 ± 4.1% (group 1) versus -19.4 ± 3.9% (group 2) versus -19.8 ± 4.0% (group 3), p<0.001).

**Conclusion:**

although global LV function, assessed with conventional echocardiographic parameters, was normal, the 2D-STI analysis showed slight but significant decrease in the global and segmental longitudinal and circumferential LV strain during the long-term follow-up after coronary arteries reimplantation.

## Introduction

Complete transposition of the great arteries (TGA) is the most common cyanotic heart disease in newborns and infants [1]. The introduction of anatomical correction of TGA with the arterial switch operation (ASO) was associated with low surgical mortality [2] and good long-term survival [3]. Furthermore, the surgical treatment of anomalous origin of the coronary artery from the pulmonary artery (ALCAPA) involving coronary reimplantation was reported to be the technique of choice and associated to lower short and long-term morbi-mortality [4]. The Ross procedure is accepted in the treatment of congenital aortic valve disease to avoid oral anticoagulation and to achieve potential valve growth [5].

A major concern about the outcome of the coronary reimplantation is still its repercussions on the cardiac function, which is a major prognostic factor. Some anomalies could develop during the follow-up such as the neoaortic root dilation and valve regurgitation [6], decreased coronary blood flow reserve [5], depressed left ventricular (LV) contractility, LV wall motion abnormalities and reversible myocardial perfusion defects [7]. Thereby, persistent subclinical cardiac impairment after surgical treatment could develop during the long-term follow-up [8,9]. The long-term cardiac worsening performance has been previously reported after surgical correction of a congenital heart defect [10,11]. However, limited and controversial data have been reported concerning the long-term cardiac function changes in patients with coronary reimplantation [4,9,12]. To our knowledge, there were no previous reports comparing several groups of operated CHD with or without coronary reimplantation as a part of surgical correction and controls to determine the even role of coronary reimplantation on cardiac performance during the long-term follow-up.

Currently, the development of echocardiographic techniques, including the tissue Doppler imaging (TDI) and the speckle tracking imaging (STI), allows a better detection of subclinical global and regional myocardial dysfunction [13,14]. The aim of the present study, as a part of the clinical follow-up, was to highlight the long-term changes in myocardial function following coronary reimplantation using TDI and STI echocardiography.

## Methods

**Study population:** this case-control study included 56 patients aged ≤4 years-old. The study was performed on patients who were followed for at least 6 months after the operation and who visited the pediatric cardiology outpatient between October 2015 and May 2016. A total of 21 patients were treated by coronary reimplantation (group 1) and 15 patients had surgical correction of cardiac septal defect (group 2). Each group was compared to 20 healthy controls (group 3). All the patients were asymptomatic at the moment of inclusion. An oral informed consent was obtained before the participation to the protocol. For all the subjects (under 18 years old of age), an oral parental consent was also obtained. Different physical parameters were recorded for all participants. The body surface area (BSA) was calculated by the Mosteller formula [15]. The 2D transthoracic echocardiography (TTE), Doppler studies and STI were also performed using a GE Vivid E9 ultrasound machine.

**Two-dimensional trans-thoracic echo cardiographic (2D-TTE) study:** the 2D-TTE was performed to assess biventricular performance using a commercially available system (vivid-E9, general electric healthcare, Horten, Norway). Images were stored in digital format to allow off-line analyses using EchoPac. Off-line analysis was performed by 1 observer to limit possible interobserver variability.

**Conventional echocardiographic and Doppler imaging study:**parasternal long-axis view was used for the M-mode measurements of left atrial diameter, interventricular septal, LV posterior wall thickness (IVST and LVPWT), LV end-diastolic and end-systolic dimensions (LVEDD and LVESD). The LV fractional shortening (LVFS) was calculated as (LVEDD - LVESD)/ LVEDD. The LV ejection fraction (LVEF) was calculated from LV volumes by the modified biplane Simpson rule and expressed as a percentage. The pulsed Doppler transmitral flow velocity profile was obtained from the apical four-chamber view by the sample volume positioned below the mitral leaflets. Different parameters were evaluated: peak transmitral flow velocity in early diastole (E), peak transmitral flow velocity in late diastole (A), E/A ratio, and the E deceleration time (DT). The tissue Doppler imaging (TDI) was also performed in the four-chamber view, with the mitral and tricuspid annular planes perpendicular to the ultrasound beam. Pulsed TD sample volume was placed at the septal and lateral aspects of the mitral annulus and at the lateral aspect of the tricuspid annulus. Measurements were made of peak systolic (S´), peak early diastolic (E´) and late peak diastolic myocardial velocities (A´) and the E/E´ ratio at the lateral mitral annulus. To assess the right ventricular (RV) systolic function, we also measured the peak systolic (S) velocity at the lateral tricuspid annulus and the tricuspid annular plane systolic excursion (TAPSE) as previously described [16].

**Speckle tracking imaging study:** the global and regional LV function was assessed by 2D- strain, expressing as percent change in segment length. Negative values represent segmental shortening, whereas positive values represent lengthening. End diastole was defined at the peak R wave of the electrocardiographic QRS complex and end-systole defined at aortic valve closure determined in the apical 5-chambers long-axis view. The following six views were displayed: the apical four-chamber view, the apical three-chamber view, the apical two-chamber view and the three short-axis views, the apex of the LV, the midlevel of the LV and the basal level of the LV. The endocardial border was drawn manually and thickness of the region of interest adjusted to cover the myocardium and exclude the pericardium. Automated tracking was performed and if necessary, manually corrected [12]. Longitudinal regional function was measured in all 6 wall regions (anteroseptal, anterior, lateral, posterior, inferior and septal) of the LV. The radial and circumferential regional function was measured in the LV at the apical, mid and basal ventricular levels [17]. Each slice was automatically subdivided in 6 segments corresponding to the ventricular wall regions analysed for longitudinal function. Longitudinal, radial and circumferential peak systolic strain for all segments were obtained and averaged to express global LV longitudinal, radial and circumferential strain.

**Statistical analysis:** statistical analysis was performed using Statistical Package for Social Sciences version 20.0 (IBM SPSS, Chicago, IL, USA). Continuous variables with normal distribution were presented as the mean (standard deviation, SD). Qualitative variables were given as percent. Correlations between continuous variables were assessed using Pearson´s or Spearman´s correlation analysis. Differences between the individual groups were tested for significance by 1-way ANOVA and the post hoc test. Univariate analysis of the effects of each continuous variable was performed with linear regression. A p-value under 0.05 was considered as statistically significant.

## Results

**Clinical characteristics:** a total of 21 patients who underwent surgical correction with coronary reimplantation were enrolled (group 1). Fifteen patients had surgical closure of atrial or ventricular septal defect (group 2) and 20 healthy controls (group 3) were enrolled. All patients were asymptomatic and none had undergone further surgery since the initial operation. In the group 1, the average age at the moment of inclusion was 11.2 ± 4.5 years. The mean follow-up duration was 8.3 ± 3.2 years. The average age of group 2 patients was 12.3 ± 4.4 years and the mean duration of the follow-up was 8.7 ± 2.5 years. Demographic characteristics of all participants are summarized in Table 1. There was no difference concerning the clinical findings between the 3 study groups. A total of 14 patients underwent ASO, 6 patients underwent Ross intervention and only 1 patient presented ALCAPA. Before ASO, 12 of 14 patients underwent a balloon atrial septostomy. In 7 of 14 patients with TGA, a ventricular septal defect was closed in addition to ASO. No difference was observed in aortic cross clamp time between patients with and without coronary reimplantation. The time of cardio-pulmonary bypass (CPB) was significantly longer in patients whose surgery included coronary reimplantation. Only 4 patients had coronary angiography control, which was normal. All the patients were in sinus rhythm without conduction abnormalities.

**Table 1:** demographic characteristics in the different study groups

	Group 1 (n=21)	Group 2 (n=15)	Group 3 (n=20)	p-value
Age (years)	11.2 ± 4.5	12.3 ± 4.4	10.9 ± 4.1	0.77
Sex (M/F)	13/8	9/6	10/10	0.45
Weight (Kg)	41.2 ± 19	43.1 ± 13	38.0 ± 20	0.61
Height (cm)	142.6 ± 23	151.6 ± 25	140.4 ± 23	0.76
BSA (m2)	1.26 ± 0.4	1.35 ± 0.4	1.20 ± 0.4	0.48
Follow-up duration (years)	8.3 ± 3.2	8.7 ± 2.5	-	0.68
Aortic clamp time (min)	76 ± 34	56 ± 22	-	0.11
CPB time (min)	131 ± 50	90 ± 26	-	0.01

All data are expressed by mean ± SD; M: male; F: female; BSA: body surface area; CPB: cardio-pulmonary bypass; min: minutes

### Echocardiographic characteristics

**MM - mode and 2D echocardiographic exam:** the MM-mode and 2D-echocardiographic data are presented in Table 2. No differences were found between the 3 groups concerning the LV and LA diameters. The LVFS and LVEF were normal in the several study groups with no statistic difference. Seven patients had trivial aortic regurgitation, 3 had mild aortic regurgitation, 5 patients had mild pulmonary regurgitation and 1 patient had moderate pulmonary regurgitation. None of the controls had aortic regurgitation. Ten patients and 8 controls had trivial mitral regurgitation.

**Table 2:** comparison of the MM-mode and 2D-echocardiographic data between the different study groups

	Group 1 (n=21)	Group 2 (n=15)	Group 3 (n=20)	p-value
LVEDD (mm/m2)	41.2 ± 4.9	40.9 ± 6.9	39.2 ± 7.0	0.30
LVESD (mm/m2)	27.1 ± 4.2	26.0 ± 5.4	26.0 ± 4.5	0.42
IVST d (mm/m2)	6.9 ± 1.3	7.2 ± 0.7	7.2 ± 2.0	0.64
IVST s (mm/m2)	8.4 ± 1.5	8.6 ± 0.9	8.2 ± 2.0	0.75
LVPWT d (mm/m2)	6.5 ± 1.9	7.2 ± 1.3	7.0 ± 1.7	0.37
LVPWT s (mm/m2)	9.6 ± 2.0	9.2 ± 1.0	8.9 ± 1.4	0.20
AoD (mm/m2)	23.2 ± 4.5	24.3 ± 5.5	22.4 ± 4.4	0.57
LAD (mm/m2)	26.9 ± 7.3	27.9 ± 6.3	25.5 ± 6.1	0.51
LVFS (%)	34.2 ± 5.2	34.4 ± 3.7	33.0 ± 5.2	0.51
LVEF (%)	60.5 ± 0.9	60.7 ± 0.8	61.1 ± 0.7	0.92
LAA (cm2)	10.7 ± 3.1	11.6 ± 2.5	9.0 ± 4.5	0.18

All data are expressed by mean ± SD; LVEDD: left ventricular end diastolic diameter; LVESD: left ventricular end diastolic diameter; IVST: interventricular septal thickness; LVPWT: left ventricular posterior wall thickness; Ao D: aorta diameter; LAD: left atrial diameter; LVFS: left ventricular fractional shortening; LVEF: left ventricular ejection fraction; LAA: left atrial area

**Doppler study:** comparison of the standard transmitral Doppler parameters yielded similar E-wave, A-wave and E/A ratio between the study groups. The deceleration E-wave time was more important in group 1 than in the two other groups (p=0.02). Comparison of TDI parameters measured from the lateral mitral annulus also demonstrated lower S´ rate in the group 1 patients as compared with each group 2 and controls group (p<0.001). The LV diastolic function as assessed by the mean E´ velocity was also lower in the 2 patients groups comparing with controls (p<0.001). But there was no difference in the E´ velocity and E/E´ ration between group 1 and group 2 (Table 3). Both parameters describing RV systolic performance, including RV S´ and TAPSE were impaired in the operated patients. The RV S´ velocity and the TAPSE were more decreased in the group of patients operated with coronary reimplantation than in the 2 other groups (Table 3).

**Table 3:** comparison of the Doppler parameters of the LV and the RV between the different study groups

	Group 1 (n=21)	Group 2 (n=15)	Group 3 (n=20)	p-value	P (group 1 vs 2)	P (group 1 vs 3)	P (group 2 vs 3)
	Mitral inflow						
E (cm/s)	103.2 ± 17.7	105.6 ± 20.1	104.3 ± 15.6	0.92	0.75	0.93	0.86
A (cm/s)	60.1 ± 14.1	60.6 ± 20.9	65.0 ± 15.0	0.59	0.98	0.29	0.31
E/A	1.76 ± 0.3	1.79 ± 0.2	1.64 ± 0.2	0.31	0.47	0.11	0.09
DT (ms)	202.0 ± 46	183.0 ± 29	168.0 ± 37	0.02	0.16	0.02	0.13
	**TDI in the mitral annulus**						
Lateral S'(cm/s)	8.6 ± 1.3	11.5 ± 1.9	11.6 ± 1.9	<0.001	<0.001	<0.001	0.79
Septal S' (cm/s)	10.6 ± 1.3	8.5 ± 1.3	9.7 ± 1.4	0.76	0.19	0.77	0.59
E' (cm/s)	16.3 ± 3.0	16.4 ± 2.5	21.5 ± 3.1	<0.001	0.68	<0.001	<0.001
E/E'	6.5 ± 2.1	6.5 ± 1.9	4.89 ± 0.7	0.004	0.87	0.01	0.02
	**RV systolic function**						
S' Tricuspid annulus (cm/s)	8.4 ± 1.6	10.7 ± 1.1	14.3 ± 1.3	<0.001	<0.001	<0.001	<0.001
TAPSE (mm)	14.8 ± 1.9	17.6 ± 2.3	20.5 ± 2.7	<0.001	0.004	<0.001	0.004

All data are expressed by mean ± SD; A: peak transmitral flow velocity in late diastole; DT: deceleration time; E: peak transmitral flow velocity in early diastole; E': peak early diastolic myocardial velocity; S': peak systolic myocardial velocity; TAPSE: tricuspid annular plane systolic excursion; TDI: tissue Doppler imaging; RV: right ventricle

**Speckle tracking imaging:** the LV global peak strain parameters revealed a significant decrease in the longitudinal and circumferential strain components. The LV global longitudinal strain (GLS) values were lower in both groups of operated patients than controls (-19.9 ± 2.2% (group 1) versus -20.9 ± 1.6% (group 2) versus -22.9 ± 2.3% (group 3), p<0.001). The patients with coronary reimplantation had the lowest values. But there was no difference between the 2 groups of operated patients with or without coronary reimplantation (Table 4). The LV global circumferential strain was also decreased in the group 1 patients as compared with the 2 other groups (-16.6 ± 4.1% (group 1) versus -19.4 ± 3.9% (group 2) versus -19.8 ± 4.0% (group 3), p<0.001). The LV GCS values were similar between the patients operated for cardiac septal defect and controls (Table 4). The LV global radial strain (GRS) was also similar in the different study groups (Table 4).

**Table 4:** comparison of the LV global strain parameters between the different study groups

	Group 1 (n=21)	Group 2 (n=15)	Group 3 (n=20)	P	P (Group 1 vs Group 2)	P (Group 1 vs Group 3)	P (Group 2 vs Group 3)
GLS	-19.9 ± 2.2	-20.9 ± 1.6	-22.9 ± 2.3	<0.001	0.36	<0.001	0.01
GCS	-16.6 ± 4.1	-19.4 ± 3.9	-19.8 ± 4.0	<0.001	<0.001	<0.001	0.53
GRS	34.0 ± 16.2	36.8 ± 14.1	37.0 ± 19.7	0.27	0.24	0.18	0.31

All data are expressed by mean ± SD; LVGCS: left ventricular global circumferential strain; LVGLS: left ventricular global longitudinal strain; LVGRS: left ventricular global radial strain

**Figure 1:**
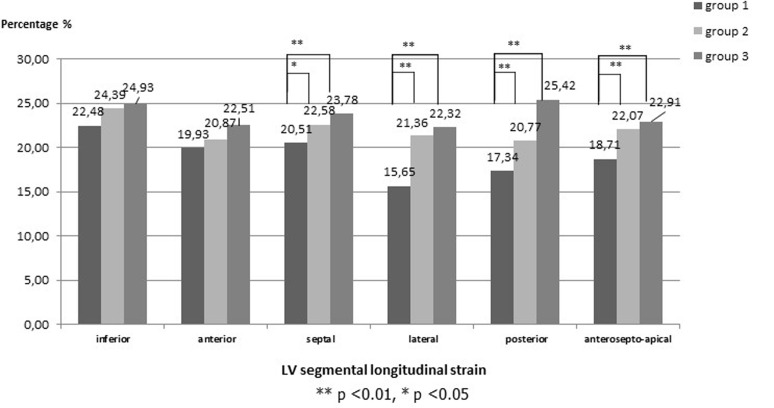
segmental longitudinal strain in the different left ventricular (LV) walls

**Figure 2:**
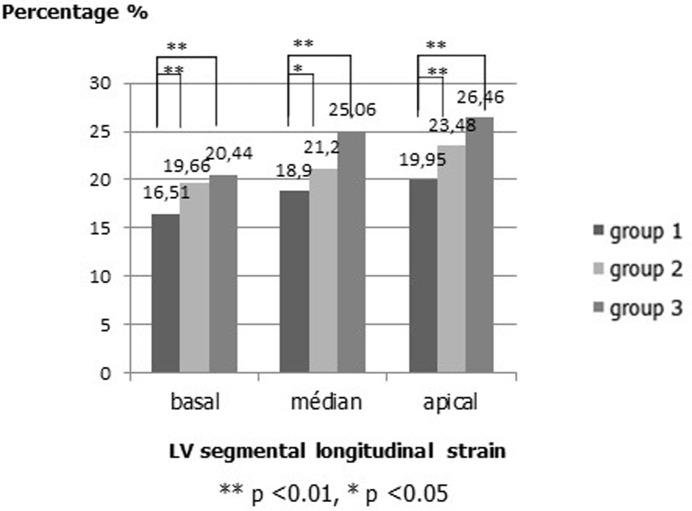
segmental longitudinal strain in the different left ventricular (LV) levels

In addition to global peak strain parameters, segmental peak strain parameters were compared in patients versus controls. After coronary reimplantation, the longitudinal strain was significantly decreased in the posterior, lateral and anteroseptal walls as compared with the other patients and controls (posterior: -17.34 ± 2.4% (group 1) versus -20.77 ± 2.6% (group 2) versus -25.42 ± 2.3% (group 3); lateral: (-15.65 ± 2.2% (group 1) versus -21.36 ± 1.9% (group 2) versus -22.32 ± 2.1% (group 3); anteroseptal (-18.71 ± 3.1% (group 1) versus -22.07 ± 2.6% (group 2) versus -22.91 ± 2.2% (group 3); all p<0.001) (Figure 1). There was no significant difference in the other LV walls. The longitudinal strain values from all 6 LV wall regions were averaged for basal, mid and apical levels. In patients, longitudinal strain was slightly decreased at all ventricular levels, most pronounced at the basal level (Figure 2). The all LV levels strain values was notably the most decreased after coronary reimplantation (Figure 2).

**Figure 3:**
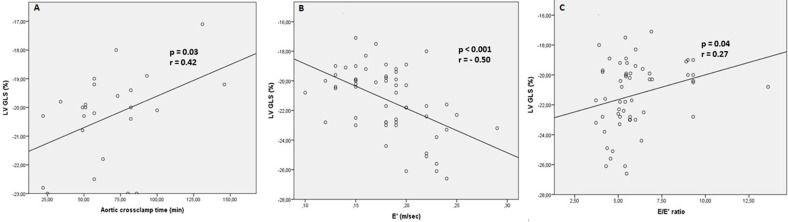
A) correlation between the LV GLS and the aortic cross clamp time; B,C) correlation between the LV GLS and the E' velocity and the E/E' ratio

A significant correlation was observed between the aortic cross clamp time and the LV GLS at the long-term follow-up (p=0.03 and r=0.42) (Figure 3A). There were also significant correlations between the lateral LV S´ velocity and the LV GLS (p=0.01 and r=-0.34) and the diastolic LV function parameters (E´, DT and E/E´) and the LV GLS (Figure 3B, C. Correlations between LV GLS and clinical and echocardiographic findings are summarized in Table 5. Finally, the age at operation, the CPB time and the aortic cross clamp time were not correlated to the RV performance parameters at the long-term follow-up.

**Table 5:** correlation between the LV GLS and the other parameters in the study population

	CPB time	Ao cross clamp time	Age at the surgery	LV S'	E'	E/E'	DT	TAPSE	RV S'
r	0.38	0.42	0.16	-0.34	-0.50	0.27	0.39	-0.36	-0.52
p	0.05	0.03	0.46	0.01	<0.001	0.04	0.002	0.006	<0.001

Ao: aortic; CPB: cardio-pulmonary bypass; DT: deceleration time; E: peak transmitral flow velocity in early diastole; E': peak early diastolic myocardial velocity; S': peak systolic myocardial velocity; TAPSE: tricuspid annular plane systolic excursion

## Discussion

In this present study, the operated patients with coronary reimplantation presented a slight but significant decrease in the LV GLS and the LV GCS at the long-term follow-up as compared with each group of operated patients for isolated cardiac septal defect and controls. The segmental longitudinal strain was decreased in all LV walls at the long-term follow-up after cardiac surgery. This decrease was more pronounced after coronary reimplantation and reached preferably the posterior, lateral and anteroseptal LV walls and the basal levels. This was not revealed by the conventional LV function parameters (LV EF and LV FS), which were within the normal range values in the different groups of the study population. The LV diastolic function was also normal, except for DT and E´ velocity, which were significantly decreased in the patients. Thereby, the assessment of myocardial deformation by the STI may contribute to early detection of subclinical LV impairment in the follow-up of these patients. In addition, the RV systolic function remained impaired during the follow-up.

The data describing the long-term cardiac performance changes after coronary reimplantation and cardiac septal defects surgery are quite limited and the echocardiographic findings are still conflicting. Although asymptomatic, the follow-up of patients with coronary reimplantation, TGA after ASO and Ross intervention, is still characterized by decreased exercise capacity, peak VO2 [8,18,19]. Hemodynamic restrictions, including perfusion defects, reduced myocardial blood flow and coronary reserve [5] and then impaired cardiac performance [9], could add to this. Similar to our findings, Pettersen *et al*. [12] in a study about 22 patients with TGA reported a slight decrease in the LV longitudinal shortening as compared with healthy controls. Although standard measurements of global systolic LV function were normal in patients with TGA, the longitudinal strain was decreased in all ventricular regions and most marked in the apical segments. Contrary to our results, the LV circumferential shortening was similar in the 2 groups [12]. Klitsie *et al*. [9] showed that there was no difference concerning the LV performance during the mid-term follow-up between TGA group and controls. The authors reported that the LV function recovered to the normal values within the first postoperative year if early ASO is performed. In contrast, the RV systolic and diastolic function remained impaired during the follow-up.

A study by Di-Salvo *et al*. [20] demonstrated a significant decline in the longitudinal myocardial deformation, despite a normal LV ejection fraction, in a large series of 62 asymptomatic ASO patients by using STI. The segmental longitudinal deformation was significantly impaired in the anterior and both anterior and posterior septal walls. However, the global circumferential strain and LV torsion were similar to controls [20]. In this population, the reduction of longitudinal strain was significantly correlated with the age at surgical repair [20]. Grotenhuis *et al*. [21] also demonstrated decreased global systolic LV function by magnetic resonance imaging at 16-year follow-up after ASO, suggesting that our findings, such as previous studies, might represent a slight beginning dysfunction that eventually may become apparent by standard measurements of the LV function. The RV conventional Doppler flows, tissue Doppler flows and LV performance were also impaired during the short-to mid-term follow-up of the pediatric patients who underwent ASO [22].

The echocardiographic data during the long-term follow-up in patients with atrial and ventricular septal defects (ASD, VSD) are also still controversial. Several previous studies reported a decrease in the cardiac performance immediately after surgery and a varying degree of the LV function recovery [10]. After ASD surgery, studies describing long-term follow-up, using the TDI velocities, generally reported complete recovery of the LV performance to the normal range values [23]. One previous study, which used TDI-derived LV strain and strain rate measurements, reported a regional impairment in the LV longitudinal performance in all the studied segments that persist after the first postoperative year [24]. Furthermore, the RV function, as assessed using both TDI and strain imaging, was still impaired several years after surgical ASD closure [23,24]. After VSD surgery, several studies had reported an immediate postoperative decrease in the LV systolic and diastolic performance [25-27] and a progressive recovery to normal values during the follow-up [28]. Studies describing more long-term follow-up of ventricular function in VSD patients are limited. A previous study described decreased LV S´ and E´ 11 years after surgery [29]. Another study reported persistent global and regional LV strain after surgical repair of multiple VSD and the impairment was proportional to the patch repair area [30].

As in ASD patients, decreased RV systolic function was observed in VSD patients immediately after surgery [28,31]. But there were debatable findings of the RV function at the long-term follow-up. In one study, there was a recovery to normal values within the first postoperative month [28]. These results contrast with results of another study in VSD patients that described significantly lower TAPSE and RV S´ measurements in patients versus controls after surgery [11]. The different mechanisms of the long-term LV function deterioration after cardiac surgery, including coronary reimplantation or not, are still poorly explained. There were no previous studies comparing the long-term cardiac performance changes after septal defects surgical repair and coronary reimplantation as a part of surgical treatment. In fact, the alteration of the LV longitudinal deformation after coronary reimplantation could be multifactorial. The ischemia and then the myocardial dysfunction could result from surgical repair involving coronary reimplantation and circulatory arrest.

Previous studies have reported a reduced flow reserve that could alter the myocardial perfusion and the cardiac contraction [5,7]. Furthermore, ischemia might be the result of a hypoplastic left anterior descending coronary artery, which have been described in many patients TGA [5]. Because of the abnormal LV strain values in the 2 different groups of operated patients, we could also speculate that the cardiac surgical procedure itself might alter the cardiac performance. In addition, the decrease in the LV longitudinal strain might be due to differences in the myocardial architecture between patients and controls [12]. In fact, the potential differences in fiber structure at the base might be due to the altered relation between the ventricular outlet parts [12]. Our study supports this hypothesis, as it demonstrated that decrease was most pronounced at the basal levels. We have also demonstrated that although the reduction in the LV strain might be due in part to the open-heart surgery, the coronary reimplantation was associated with a marked LV strain impairment, suggesting additional adverse mechanisms that could not be totally elucidated. Thereby, these patients could be closely monitored.

Finally, our findings were concordant with the previous one that yielded persistent RV dysfunction after cardiac surgery. We also demonstrated that the RV systolic decrease was more marked after coronary reimplantation. The precise mechanism could not be totally elucidated. In fact, the RV persistent impairment might be attributable to different causes, such as the surgical trauma, durable hypoxia [32], ischemic and inflammatory cascades secondary to incomplete myocardial protection and cardio-pulmonary bypass [33] or the damage of the coronary arteries [12]. Despite previous studies reported significant correlation between the postoperative RV performance and the CPB and the aortic cross clamp time [27,34], there was no correlation in the present study.

**Study limitations:** a limitation for our study was the relatively low case number of each group; the finding of decreased longitudinal strain should be correlated to coronary angiographic findings. Yet, coronary angiography was not clinically indicated for all patients and was thus made impossible for ethical reasons; we only used 2D STI analysis, whereas, it was recently reported the added value of 3D strain; the finding of STI analysis could be, ideally, correlated to the MRI findings. But, MRI is expensive and not always available for routine monitoring.

## Conclusion

Although global LV function, assessed with conventional echocardiographic parameters, was normal, the 2D-STI analysis showed slight but significant decrease in the global and segmental longitudinal and circumferential LV strain during the long-term follow-up of different CHD patients. The strain decrease was more pronounced after coronary reimplantation. The RV systolic function remained also impaired in these patients during the follow-up. These findings highlight the importance of long-term follow-up after CHD surgery and especially if associated with coronary reimplantation.

### What is known about this topic

- The conventional echocardiographic study during long-term follow-up after cardiac surgery is important to detect slight changes in the biventricular function even in asymptomatic patients;
- The speckle tracking echocardiography is a relatively a new non-invasive imaging technique that allows an objective assessment of the regional and global myocardial function.

### What this study adds

- The conventional echocardiography can detect some differences after coronary reimplantation but it is incapable to reveal differences in intrinsic myocardial functions. Thereby, the evaluation of STI can be useful for identifying previously undetected differences in these patients. The present study showed a lower LV global and segmental longitudinal and circumferential strain after coronary reimplantation as compared with cardiac surgery for isolated septal defects and healthy controls;
- Considering our results and the large heterogeneity of echocardiographic pattern generally found in operated CHD, several possible clinical applications of deformation parameters approach can be advised.
